# Comparison of greater occipital nerve and greater occipital nerve + supraorbital nerve block effect in chronic medication overuse headache

**DOI:** 10.3906/sag-2009-101

**Published:** 2021-06-28

**Authors:** Nermin TEPE, Oktay Faysal TERTEMİZ

**Affiliations:** 1 Department of Neurology, Faculty of Medicine, Balıkesir University, Balıkesir Turkey; 2 Neuropsychiatry Centre, Health Sciences Institute, Gazi University, Ankara, Turkey3Department of Algology, İzmir University of Health Sciences, Tepecik Training And Research Hospital, İzmir Turkey; 3 Department of Algology, İzmir University of Health Sciences, Tepecik Training And Research Hospital, İzmir Turkey

**Keywords:** Medication overuse headache, peripheral nerve blocks, greater occipital nerve, supraorbital nerve block

## Abstract

**Background /aim:**

In this study, we aimed to compare the efficacy of greater occipital nerve (GON) block alone and GON combined with supraorbital nerve (SON) block in the treatment of medication overuse headache (MOH).

**Material and methods:**

82 patients were divided into two groups: 41 patients were administered bilateral GON block while the other 41 patients GON + SON block. Nerve blocks were administered every 10 days for a total of 5 sessions. After each administration and 20 days after the last injection, information on pre and post treatment numerical rating scale (NRS) score, number of painful days, analgesic intake, duration of pain were collected.

**Results:**

The decrease in headache evaluation parameters was similar in both groups after the block. The NRS scores in the GON and GON + SON groups before the treatment was (8.2 ±  0.7, 8.5  ± 0.7), the number of painful days in a month was (21.4 ±  6.9, 21.2 ± 4.6 days), the number of analgesics taken monthly was (45 ±  25.6, 47.5 ±  29.9), the duration of pain was (44.9 ±  24.6, 41.7 ±  22.8 h), respectively. On the 60th day of treatment, the NRS scores in the GON and GON + SON groups were found to be (6.8 ±  2.5, 4.8 ± 2.3), the number of painful days in a month was (4.2 ±  3.3, 2.2 ±  1.5), respectively. The number of monthly nalgesic consumption was (4.4 ±  3.8, 0.9 ±  1.2), and the duration of pain was (28.4 ±  19.3, 19.4  ± 16.1 h).

**Conclusion:**

This study showed significant reductions in headache parameters in both groups. However, NRS score, analgesic intake, number of painful days, and pain duration significantly better improved in the GON block added SON block group.

## 1. Introduction 

Medication overuse headache (MOH) is one of the causes of headaches due to the excessive use of analgesics, triptans, and ergotamins in patients with primary headaches, such as migraines or tension-type headaches. In a prevalence study conducted in our country, the prevalence was found to be 2.1% in society and 8.2% in individuals with migraine [1]. In ICHD-3 (International Classification of Headache Disorders) published in 2018, MOH it is classified under the title of secondary headaches, and it is described as a new type of headache that occurs due to medication overuse by a patient with a primary headache or a significant deterioration in the previous headache type. The patient can be diagnosed as a result of using at least 10 tablets per month for triptan, ergotamine, and opioids, and/or at least 15 tablets per month for acetylsalicylic acid, paracetamol, and nonsteroidal anti-inflammatory drugs for longer than three months [2]. Risk factors for relapse of MOH are mainly the type of primary headache, type of analgesic, combined psychological disorders, socioeconomic factors (marital status, unemployment, smoking, alcohol intake), and clinical features of headache (severity and disease process) [3]. 

Peripheral nerve blocks can also be used in primary headaches, secondary headaches, and cranial neuralgias. Peripheral nerve blocks in headaches involve greater occipital, lesser occipital, supratrochlear, supraorbital, and auriculotemporal nerve injections, and its technical success depends on cutaneous anesthesia. The greater occipital nerve (GON) originates from the dorsal ramus of C2 as well as the C3 segments of the spinal cord and contains only sensory fibers. Sensory input from the ophthalmic branch of the trigeminal nerve and GON are thought to be transmitted to the trigeminal nucleus caudalis. GON block decreases afferent input to trigeminal nucleus caudalis resulting in attenuated neuronal hyperexcitability and modulation in central pain [4]. A study revealed an 85% positive response over 6 months in GON and supraorbital nerve (SON) block and showed that treatment had a long-term effect [5]. In chronic migraine, GON blockage can be performed only with local anesthesia or with a combination of steroid-local anesthesia. However, the mechanism of action remains unclear and the duration of the block varies individually. Their analgesic effects are beyond the duration of anesthesia typically caused by nerve block and provide a painless period for some patients for several weeks or even months. There is no standardized approach for drug dosage, injection volumes, and number of sessions [6–9]. In 2013, the American Headache Society published recommendations for the implementation of the peripheral nerve block, including the GON block for headache [10].

In this study, we aimed to investigate the difference in efficacy between using only GON block and applying GON block and SON block together in the treatment of medication overuse headache.

## 2. Material and methods

### 2.1. Study population

This study is a retrospective study and was gathered from hospital records of MOH diagnosed patients who had undergone GON and GON combined SON block. Ethical Committee approval was obtained from the Clinical Research Ethics Committee of Balıkesir University Faculty of Medicine (2018/148). A total of 313 patients were screened for the study between September 2015 and June 2018. A total of 82 patients who complied with the study criteria were included in the study. 41 patients were administered bilateral GON block and 41 were administered bilateral GON added to SON block. These nerve blocks were not performed on individuals with history of allergy to local anesthetics, history of malignancy, history of cervical or cranial surgery, history of bleeding diathesis, history of major psychiatric disorders (major depression, etc.), patients with neuromuscular dysfunction, patients with uncontrolled hypertension, hypothyroidism or hyperthyroidism, patients with open skull defect, patient on anticoagulants. Patients with other chronic painful syndromes were excluded.

Injections were administered by the same pain physician. 3 mL (7.5 mg) 0.25% bupivacaine was administered for bilateral GON block and 1 mL (2.5 mg) 0.25% bupivacaine for the bilateral SON block. Nerve blocks were administered every 10 day for total 5 sessions. NRS score, number of painful days, analgesic consumption, and duration of pain were screened from patient files. GON block was administered subcutaneously to the lateral 1/3 of the occipital protrusion on a line from the occipital protrusion to the mastoid process using a 26 gauge (G) 13 mm needle. Supraorbital nerve block was performed after palpating the corrugator muscle in the midline of the pupil at the upper edge of the orbit, a 26 G 13 mm needle was advanced laterally and injected at a slight angle to prevent it from entering the foramen. The patients were evaluated during 5 injections administered an average of 10 days apart. In addition, all patients were reassessed 20 days after the last injection.

### 2.2. Statistical analysis

SPSS for Windows Release 22.0 software (IBM Corp., Armonk, NY, USA) was used. Chi-square was used for the comparison of the qualitative data, for the comparison of the data obtained with the measurement, the Kolmogorov–Smirnov test was used to measure normal distribution, the Student’s t-test for those with normal distribution suitability, and the Mann–Whitney U test for those who do not have normal distribution suitability. ANOVA and Friedman tests were used to determine the statistical significance of the difference between the groups for variables that were not normally distributed in meeting repetitive measurements from the beginning. Paired comparisons were also used to determine the statistical significance of the difference between the groups with the Wilcoxon signed-rank test and the post-hoc Bonferroni test. Data obtained by measurement were expressed as mean standard deviation, and data obtained by count are expressed in %. The significance level was taken as P < 0.05

## 3. Results

The mean age group in the GON + SON blockade group was 42.4 ± 8.1, while it was 43.9 ± 11.3 in the GON group. No significant difference was found between the two groups in terms of demographic data shown in Table. In the GON group, 17 patients were using amitriptyline, 17 patients were using duloxetine, 4 patients were using venlafaxine and 3 patients propranolol, while, in the GON added SON group, 19 patients were using amitriptyline, 15 patients were using duloxetine, 5 patients were using venlafaxine, and 2 patients were using propranolol. These drugs were continued as a preventive treatment in patients. 

**Table:** Demographics and pain characteristics of the groups before the treatment as mean ± standard deviation. GON: Greater occipital nerve block, GON + SON: Greater occipital nerve and supraorbital nerve block.

	GON block(mean ± std dev.)	GON+ SON block (mean ± std dev.)	P < 0.05
Age	43.9 ± 11.3	42.4 ± 8.1	0.47
Weight (kg)	68.2 ± 9.4	70.2 ± 10.2	0.37
Height (cm)	165 ± 4.5	166.3 ± 7.6	0.35
NRS	8.2 ± 0.7	8.5 ± 0.7	0.13
Painful days/month	21.4 ± 6.9	21.2 ± 4.6	0.86
Analgesic intake/month	45	47.5	0.69
Attack pain duration (hours/day)	44.9	41.7	0.55

**Figure 1:**
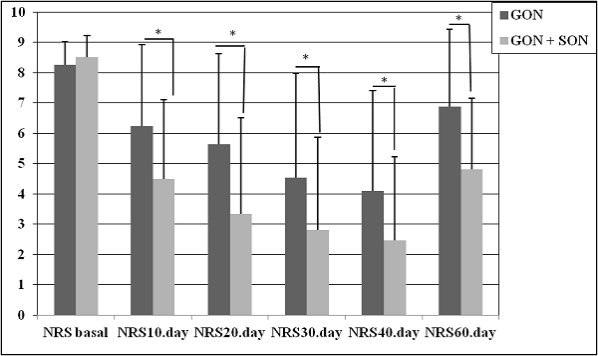
Comparison of NRS scores of both groups before and after treatment; While there was no difference between the two groups before the treatment, NRS scores significantly decreased in both groups in each administration with a 10-day interval compared to before the treatment, but this significance was found to be more evident in GON + SON (P < 0.05). GON: Greater occipital nerve block, GON + SON: Greater occipital nerve and supraorbital nerve block, NRS: Numerical rating scale.

**Figure 2:**
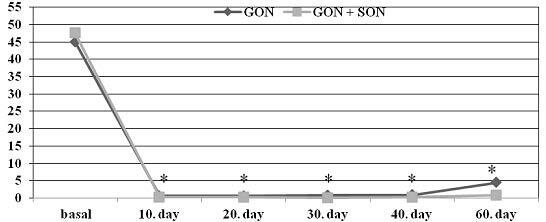
Analgesic intake amount of both groups; a significant decrease in terms of statistical significance compared to baseline was found in both groups, and this state of well-being continued for five administrations with 10-day intervals for two months (administrations were made on baseline, day 10, day 20, day 30, day 40) (P < 0.05). GON: Greater occipital nerve block, GON + SON: Greater occipital nerve and supraorbital nerve block.

**Figure 3:**
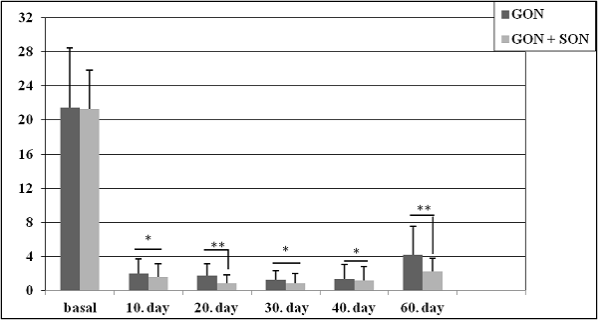
Number of painful days in both groups; statistically significant decrease was observed in both groups in the five administrations following the first administration and the baseline continuing on day 60 afterward, while this state of well-being was found to be more evident in GON + SON on day 20 and day 60 (*P < 0.05, **P < 0.01). GON: Greater occipital nerve block, GON + SON: Greater occipital nerve and supraorbital nerve block.

**Figure 4:**
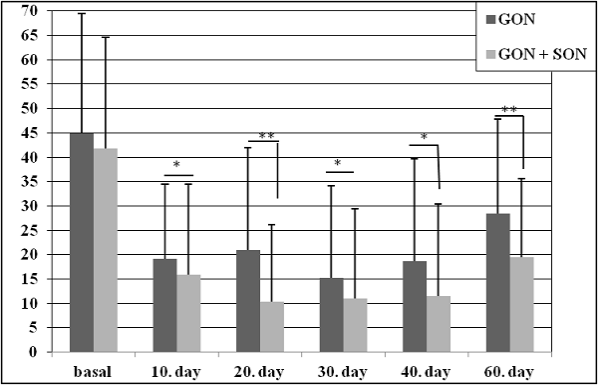
Attack pain duration (hour) of both groups; when compared to baseline in both groups, it was found that there was a statistically significant reduction in the duration of pain in five administrations with 10-day intervals and the follow-up on day 60. Besides, this decrease was found to be more evident in the GON + SON group on day 20 and day 60 (*P < 0.05, **P < 0.01). GON: Greater occipital nerve block, GON + SON: Greater occipital nerve and supraorbital nerve block.

While the NRS score before the treatment was 8.2 ± 0.7 in the GON group, it was 8.5 ± 0.7 in the GON + SON group as shown in Figure 1. The number of analgesics taken monthly was 45 ± 25.6 and 47.5 ± 29.9 in the GON and GON + SON groups, respectively as shown in Figure 2. The number of monthly painful days was 21.4 ± 6.9 in the GON group, 21.2 ± 4.6 days in the GON + SON group as shown in Figure 3. The pain duration of each attack was found to be 44.9 ± 24.6 h in the GON group and 41.7 ± 22.8 h in the GON + SON group, and there was no statistically significant difference. However, statistically significant decrease was found in terms of NRS, analgesic consumption, the number of painful days, and the duration of each attack in both groups. Furthermore, statistically significant decrease in pain duration was found only in the GON added SON group on day 20 and day 60 (P < 0.01) as shown in Figure 4 There were no life-threatening complications in either group. There were no complications other than bleeding at the injection site and pain during injection.

## 4. Discussion

In our study, both GON block and GON added SON block revealed a statistically significant decrease in NRS, analgesic consumption, painful days and attack duration; the GON added SON provided a statistically significant decrease in the attack pain duration compared to GON on day 20 and day 60, and we found that adding GON to SON was more effective in reducing the NRS score. This suggests that, in clinical practice, the GON added SON should be considered as a priority compared to GON, but a bilateral GON block can also be used. In addition, although the increase was observed in NRS, analgesic intake, painful days, and pain duration in both groups on the day 60 follow-up, it was observed that the effectiveness of the treatment continued when compared with baseline values. 

This study is the first since there has been no previous study comparing GON and GON + SON blockade in patients with (MOH). Although the American Headache Society made suggestions about the peripheral nerve blocks, doses, frequency, and amounts that may be performed in headache in 2013, there is still no clear consensus on this subject due to lack of randomized controlled trials with large number of patients and different doses and applications in different centers and branches. However, the studies from different centers showed that peripheral nerve blocks are especially effective in primary headache patients, but is not known exactly whether single or multiple blocks can be used in clinical practice since there are no studies comparing peripheral nerve blocks. In this study, we compared GON and GON added SON blocks in patients with MOH as a guide to clinical practice since there are more studies on effectiveness of GON block. 

The type of primary headache is an independent risk factor for MOH relapse, and patients with migraine tend to relapse more than patients with other types of primary headaches. The most effective and successful treatment in these patients is the discontinuation of analgesics and detoxification. It is suggested that peripheral and central sensitization is facilitated as a result of increased excitability in cortex and trigeminal ganglion in medication overuse, and supporting this idea, we may encounter a picture of perceiving painless stimulation as painful, which we call allodynia, in this group of patients as well as excessive sensitivity to sound and light as a result of increased sensitivity. In functional magnetic resonance imaging (fMRI) studies, insufficiency has been shown in descending inhibition pathways [3,11–12].

Although the exact mechanism of action of the GON block is unknown, it is thought to modulate brain excitability at the brainstem level. Cervical stimulation has been shown to directly increase brain serotonin, and in the neuropathic rat model, stimulation of spinal 5-HT receptors contributed to the reduction of pain as a result of spinal cord stimulation [5]. The recommended mechanism for the effect of the GON block on reducing migraine symptoms is thought to be the result of the link between the ophthalmic branch of the trigeminal nerve of the modulation of the afferent pathway to the trigeminal nucleus caudalis and the greater occipital nerve. With local anesthetics and corticosteroid administration, sensorial input to trigeminal nucleus caudalis decreases. In the study on rats, a functional connection was reported between the caudal trigeminal nucleus and the upper cervical segments in cranial nociception where neurons in the C2 dorsal horn receive messages from the dura, cervical cutaneous nerves, and muscles [10]. Some studies demonstrated that the GON block can significantly reduce pain severity and the number of painful days in migraine patients [13–14]. In the metanalysis, GON block significantly decreased pain score, number of headache days, and consumption of analgesics in patients with migraines compared to the control group, but it was found ineffective in the headache duration in four weeks [15]. Caputi and Firetto administered a maximum of 10 blocks with 0.5% bupivacaine for GON and supraorbital nerves in 27 migraine patients and observed an 85% benefit effect for six months. They suggest that all individuals with migraines have continuous mechanical hyperalgesia during the interictal phase, and therefore central hypersensitivity due to sensitization of extracranial perivascular nociceptors and that the course to be followed is eliminating the area of active nociception with local anesthetics, thus decreasing mechanical hyperalgesia, decreasing nociceptor sensitization, and continuing the injections until neuronal sensitivity resumes to normal [16]. Since there is no significant difference in headache duration and headache attack frequency in patients already having prophylactic drugs, adding prophylactics to the GON block is not recommended [17]. Since the optimal dose and local anesthesia for GON block in migraine patients is still unknown, 1%–2% lidocaine (10–20 mg/mL) and/or 0.25–0.5% bupivacaine (2.5–5 mg / mL) is recommended [18]. 

The limitations of our study include small sample size, lack of randomized controlled studies, and lack of longer follow-up.

In conclusion, peripheral nerve blocks are also used as a temporary treatment option in medication overuse headaches. Typically, the duration of the therapeutic effect varies between patients, but it should be done with at least 2–4 weeks intervals for patients with migraine since their benefits usually range from days to weeks. Both GON block and GON + SON block provide effective treatment in chronic analgesic headache. However, the addition of the SON block to the GON block seems to be more effective in reducing NRS, analgesic intake, the number of painful days, and pain duration.

We think that multiple nerve block will provide significant support for practice and literature because it is shown to be more effective in isolated MOH; it especially supports the patients with peripheral nerve blocks during detoxification and/or until the prophylactic drugs are started to be taken. By this way, the quality of life of the patients improves, also the number of emergency visits decreases and getting re-addicted to analgesics is prevented. Controlled randomized clinical studies to be conducted on the subject may reveal the level of evidence for GON and SON block in MOH and ensure that it is included in treatment protocols.

## Informed consent

The study protocol received institutional review board approval from ethical committee of the Balıkesir Medical Faculty (2018/148), and all participants provided informed consent.
